# Improvement in CRT: new strategies, better choices

**DOI:** 10.1007/s12471-014-0589-x

**Published:** 2014-08-21

**Authors:** E. E. van der Wall

**Affiliations:** Interuniversity Cardiology Institute of the Netherlands (ICIN) - Netherlands Heart Institute (NHI), P.O. Box 19258, 3501 DG Utrecht, the Netherlands

Cardiac resynchronisation therapy (CRT) emerged more than a decade ago as a useful form of device therapy for heart failure associated with abnormal ventricular conduction indicated by a wide QRS complex [1, 2]. According to the Guidelines of the European Society of Cardiology (ESC) entitled *Cardiac Pacing and Cardiac Resynchronization Therapy* (ESC Clinical Practice Guidelines, 2013), CRT has currently a Class I indication in patients with 1) chronic heart failure (NYHA Class II-IV despite optimal medical treatment), 2) a left bundle branch block (LBBB) on the ECG with a QRS width >120 ms (preferably >150 ms), and 3) a left ventricular ejection fraction < 35 % [3]. The magnitude of CRT benefit increases in females, in patients with non-ischaemic cardiomyopathy, and in patients with a wide QRS complex: the longer the QRS duration the more favourable the response.

CRT consists of biventricular pacing with or without an internal cardioverter defibrillator (ICD): in combination with an ICD, CRT is called CRT-D (efibrillator); without an ICD, the treatment is called CRT-P (acing). According to Daubert et al. [4], the prescription of CRT-D is currently restricted to patients in need of secondary prevention of ventricular arrhythmias or, for primary prevention, in younger patients without major concomitant illnesses. The preferential choice of CRT-P for the remainder of the ambulatory patients in NYHA class III or IV is currently acceptable. Because of insufficient data regarding the performance of CRT-P in patients presenting in NYHA functional class I or II, CRT-D is currently the device of choice for this sub-population.

In the choice between CRT-D and CRT-P, the 2013 ESC guidelines - endorsed by our national society, the NVVC- clearly state: *Owing to the potential incremental survival benefit of CRT-D over CRT-P, the prevailing opinion among the members of this Task Force is in favour of a superiority of CRT-D in terms of total mortality and sudden death. However, no strict recommendations can be made, and the Task Force prefers to merely offer guidance regarding the selection of patients for CRT-D or CRT-P, based on overall clinical condition, device-related complications and cost.*

Speaking of device-related complications and costs, these are crucial issues to be considered as there are still 20–30 % non-responders to CRT despite following the appropriate guideline indication [5–7]. Therefore, new strategies and more appropriate CRT approaches have to be implemented providing higher rates of success.

Recently, Vernooij et al. [8] from Maastricht (the Netherlands) proposed new strategies to improve the outcome of CRT. As the implantation of CRT devices comes with substantial costs, in particular CRT-D, the authors plead for a more strict and rigid application of the guidelines, or even extension of the guidelines. Outcomes from CRT can be improved by appropriate patient selection, careful positioning of right and left ventricular pacing electrodes, and optimal timing of electrode stimulation. LBBB remains the predominant substrate for CRT, and patients with this conduction abnormality yield the most benefit. However, other features, such as QRS morphology, mechanical dyssynchrony, myocardial scarring, and the aetiology of heart failure, might also determine the benefit of CRT. No single left ventricular pacing site suits all patients, but a late-activated site, during either the intrinsic LBBB rhythm or right ventricular pacing, should be selected. Positioning the lead inside a scarred region substantially impairs outcomes. Optimisation of stimulation intervals improves cardiac pump function in the short term, but CRT procedures must become easier and more reliable, perhaps with the use of electrocardiographic measures, to improve long-term outcomes. For instance, Molenaar et al. [9] showed that a 10 % increase in stroke volume can be achieved for optimisation of atrioventricular (AV) delay during exercise. In that study, a considerable number of patients showed benefit with lengthening of the AV delay during exercise.

In summary, sharper indications for CRT, and the permanent consideration of choosing CRT-P or CRT-D, would be cost-saving and might provide health benefits. This is in line with the recent findings by Looi et al. [10] who showed that CRT-D did not offer additional survival advantage over CRT-P at longer-term follow-up, as the clinical benefit of a defibrillator attenuated with time. As mentioned before, further work is therefore needed to define which subsets of patients would benefit from CRT in combination with an ICD.

Lastly, there remains an urgent need for randomised clinical trials in CRT subgroups such as in patients with right bundle branch block, NYHA Class I, atrial fibrillation, and in patients with specific conditions (pacing in children, congenital heart disease). When these studies have been completed, then we might speak of true CRT optimisation to better serve patients with chronic heart failure.
